# Food production and resource use of urban farms and gardens: a five-country study

**DOI:** 10.1007/s13593-022-00859-4

**Published:** 2023-02-01

**Authors:** Erica Dorr, Jason K. Hawes, Benjamin Goldstein, Agnès Fargue-Lelièvre, Runrid Fox-Kämper, Kathrin Specht, Konstancja Fedeńczak, Silvio Caputo, Nevin Cohen, Lidia Poniży, Victoria Schoen, Tomasz Górecki, Joshua P. Newell, Liliane Jean-Soro, Baptiste Grard

**Affiliations:** 1 University Paris-Saclay, INRAE-AgroParisTech, UMR SAD-APT, Palaiseau, France; 2 School for Environment and Sustainability, University of Michigan, Ann Arbor, MI USA; 3 Department of Bioresource Engineering, McGill University, Ste-Anne-de-Bellevue, Quebec Canada; 4 ILS Research Institute for Regional and Urban Development, Dortmund, Germany; 5 Faculty of Human Geography and Planning, Department of Integrated Geography, Adam Mickiewicz University, Poznań, Poland; 6 School of Architecture and Planning, University of Kent, Canterbury, UK; 7 Graduate School of Public Health and Health Policy, City University of New York, New York, NY USA; 8 Centre for Agroecology, Water, and Resilience (CAWR), Coventry University, Coventry, UK; 9 Faculty of Mathematics and Computer Science, Adam Mickiewicz University, Poznań, Poland; 10 University Gustave Eiffel, GERS-LEE, F-44344 Bouguenais, France; 11 IRSTV-FR CNRS 2488, Ecole Centrale de Nantes, Nantes, France; 12 University Paris-Saclay, INRAE-AgroParisTech, UMR ECOSYS, Palaiseau, France; 13 ISARA, Agroecology and Environment Research Unit, Lyon, France

**Keywords:** Urban agriculture, Urban farm, Community garden, Allotment garden, Individual garden, Sustainability, Resource use, Food-energy-water nexus, Resource efficiency, Yield

## Abstract

There is a lack of data on resources used and food produced at urban farms. This hampers attempts to quantify the environmental impacts of urban agriculture or craft policies for sustainable food production in cities. To address this gap, we used a citizen science approach to collect data from 72 urban agriculture sites, representing three types of spaces (urban farms, collective gardens, individual gardens), in five countries (France, Germany, Poland, United Kingdom, and United States). We answered three key questions about urban agriculture with this unprecedented dataset: (1) What are its land, water, nutrient, and energy demands? (2) How productive is it relative to conventional agriculture and across types of farms? and (3) What are its contributions to local biodiversity? We found that participant farms used dozens of inputs, most of which were organic (e.g., manure for fertilizers). Farms required on average 71.6 L of irrigation water, 5.5 L of compost, and 0.53 m^2^ of land per kilogram of harvested food. Irrigation was lower in individual gardens and higher in sites using drip irrigation. While extremely variable, yields at well-managed urban farms can exceed those of conventional counterparts. Although farm type did not predict yield, our cluster analysis demonstrated that individually managed leisure gardens had lower yields than other farms and gardens. Farms in our sample contributed significantly to local biodiversity, with an average of 20 different crops per farm not including ornamental plants. Aside from clarifying important trends in resource use at urban farms using a robust and open dataset, this study also raises numerous questions about how crop selection and growing practices influence the environmental impacts of growing food in cities. We conclude with a research agenda to tackle these and other pressing questions on resource use at urban farms.

**Supplementary Information:**

The online version contains supplementary material available at 10.1007/s13593-022-00859-4.

## Introduction

The environmental impacts of supplying food to cities are immense (Goldstein et al. 2017). Urban agriculture (UA) is often promoted as a means to reduce these impacts and simultaneously provide multi-functional health and well-being benefits (Gomez Villarino et al. 2021; Newell et al. 2022). UA is broadly defined as growing food in and around cities that interacts with urban areas through the exchange of materials, people, and values (Mougeot 2000). While there are many types of UA, ranging from gardens to advanced, hydroponic “plant factories,” we focus here on soil-based gardens and farms that cultivate vegetables and fruit, as these are most common (Cameron et al. 2012). An expected benefit of such systems is producing hyper-local, nutritious food for city residents. Nevertheless, growing food in cities requires water, energy, land, fertilizers, and pesticides (FAO 2011; Campbell et al. 2017; Mohareb et al. 2017) and can have negative environmental impacts. Understanding these inputs and impacts is key to ensuring that UA contributes to sustainable urban food systems.

Little is known about the quantity of food produced by and the inputs used in UA, partly due to its diversity and sometimes informal nature. UA can have both very large or small yields and can be resource efficient or inefficient, yet the key factors that drive differences are unknown (CoDyre et al. 2015; McDougall et al. 2019). An accurate understanding of UA yields and inputs, such as water, fertilizer, and compost, is essential for evaluating its potential impacts on urban resource use and local food systems as the practice expands (Cohen and Wijsman 2014). Such evaluations support more accurate projections of the amounts and types of foods consumed in cities that can be provided by UA (Weidner et al. 2019; Grafius et al. 2020), and what resources are required to support food urban production. A proper material accounting of UA would also help clarify the effect of large-scale UA on the stocks and flows of material and energy that comprise a city’s “metabolism” (Barles 2009; Van Broekhoven and Vernay 2018) and help answer critical policy questions, such as the tractability of UA as a food supply in arid, water-stressed cities. In addition, increasing knowledge of UA yields and inputs for its different forms is necessary to conduct environmental footprinting of urban food production (Dorr et al. 2021).

Data on resources used by urban farms are rarely collected because it is time consuming and often not standard farming practice (Whittinghill and Sarr 2021). Only a handful of studies provide detailed accounts of farm inputs, yields, and environmental impacts. This data gap forces researchers evaluating UA to use unrepresentative statistics from rural agriculture (McClintock et al. 2013; Aragon et al. 2019) or to estimate values for yield and input use based on secondary data (Dalla Marta et al. 2019; Weidner and Yang 2020). To study functioning UA in situ as opposed to research-oriented, ideally managed experimental urban farms, researchers use citizen science to enlist farmers to collect and report data on their farming practices (Pollard et al. 2018a).

Studies that employ citizen science frequently characterize systems qualitatively, surveying crop choices and cultivation practices, but often stop short of measuring yields and farming inputs (Algert et al. 2014; Woods et al. 2016; Kirkpatrick and Davison 2018). When such data are collected, datasets are usually limited to a relatively small number of case studies (10-35), covering one type of UA in one location (Algert et al. 2014; Pourias et al. 2015; McDougall et al. 2019; Wielemaker et al. 2019; Sovová and Veen 2020; Csortan et al. 2020). There are studies that have evaluated more than 50 cases, but these usually have a rather narrow focus on food production and do not assess resource consumption (CoDyre et al. 2015; Nicholls et al. 2020; Edmondson et al. 2020). Dobson et al. (2021) had a large sample size (163 participants) and measured a suite of indicators covering food production and resource use, but only studied one type of UA, allotment gardens.

**Fig. 1:**
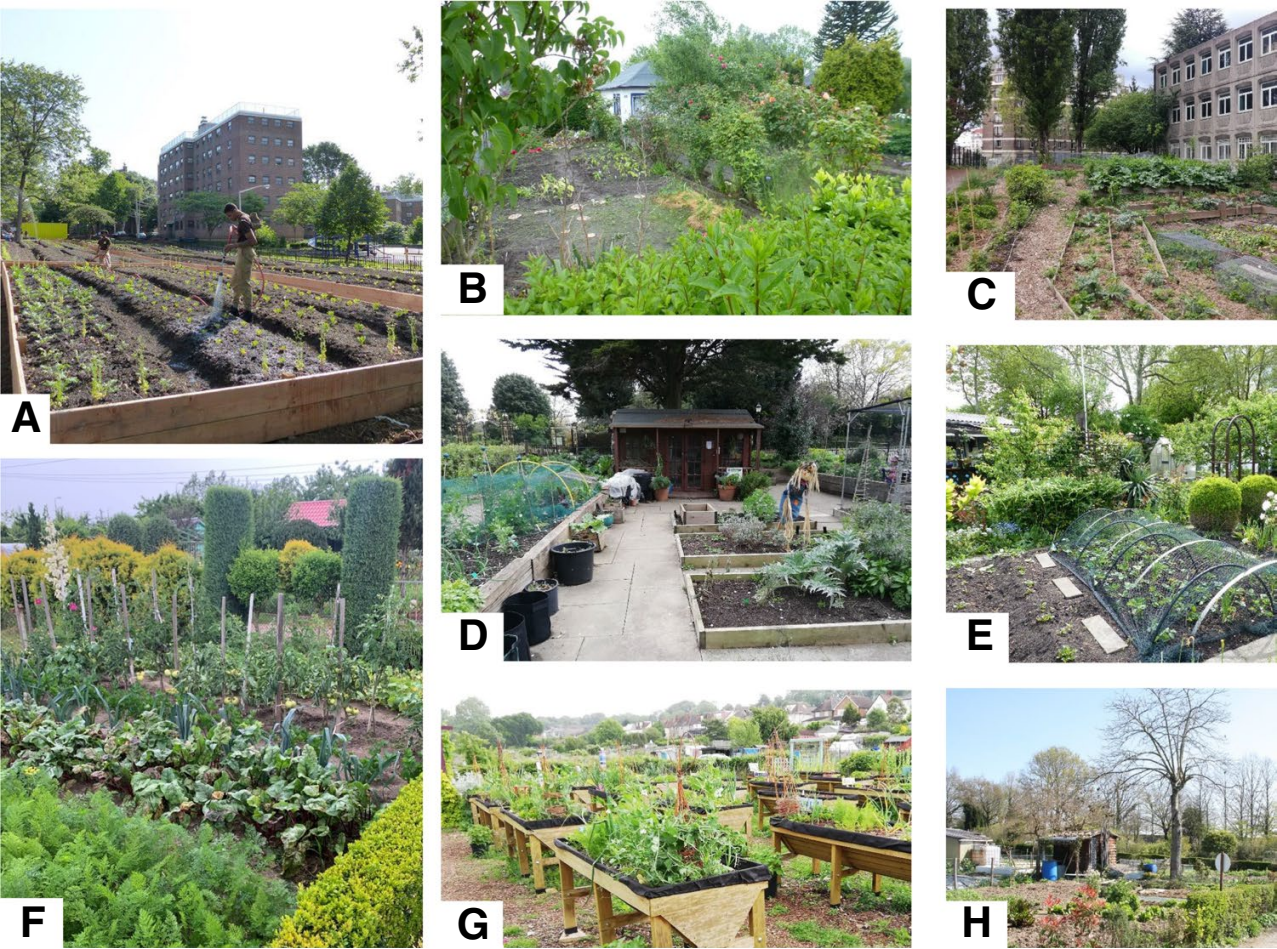
Illustration of study sites in the five countries. (**A**) Collective garden — ‘Mariners-Harbor-Farm’, New-York, USA. Source: https://greencityforce.org/service-corps/farms-at-nycha/; (**B**) Individual garden — Bochum, Germany. (**C**) Urban farms — ‘Collège Pierre Mendès France’, Paris, France. (**D**) Collective garden- UK. (**E**) Individual garden — Dortmund, Germany. (**F**) Individual garden — Gorzów Wielkopolski, Poland. (**G**) Urban farm — Mudlarks, UK. (**H**) Individual garden — ‘Les Eglantiers’, Nantes, France.

With this study, we fill this research gap by (i) measuring the level of food production and the inputs used at 72 urban farms/gardens representing three different types of UA across five countries (Fig. 1) and (ii) analyzing the patterns of food production and resources used. We measured mass and calories of food produced, the yields per crop, and crop diversity. We measured indicators of resource use including land use, irrigation water source and quantities used, type and amount of amendments such as compost and fertilizers, and energy use. With this unique dataset, we addressed the following research questions:

i. What are the land, water, nutrient, and energy demands of UA, and how and why do these demands vary?
ii. What is the yield of UA, how does it compare to conventional rural agriculture, and how does it vary across types of farms and gardens?
iii. To what degree does UA provide crop and flora diversity to cities?

To answer these questions, in the following sections, we first explain our citizen science approach and then report the findings of this research and their relation to existing literature. We conclude by examining the key decision-making implications and limitations of this study and areas where future research should extend this work.

## Material and methods

This study was carried out as part of a larger research project (called “FEW-Meter”) to understand the impacts of UA on the urban Food-Energy-Water nexus (FEW) (www.fewmeter.org). The full approach of the project and the methodology developed to measure the nexus are documented in Caputo et al. (2021). Data were collected during the 2019 growing season (March 1^ST^ to October 31^st^) using a citizen science approach in case studies in five countries: France, Germany, Poland, the UK and the USA (see Ebitu et al. 2021 for definition and discussion of citizen science in agriculture). The research was divided into four phases: (1) site selection; (2) data collection; (3) data processing; and (4) data analysis. We detail each phase below.

### Site selection 

We selected case studies using two criteria: (1) farm or garden using soil or substrate (as opposed to hydroponic or other growing system using inert medium/substrate) and (2) participant willingness to contribute to a citizen science study. Data were collected from 72 sites, which are presented in detail in Table 1. The sites correspond to three UA types:

- Nine urban farms, defined as productive spaces led by farmers with multiple goals (especially food production but also social and environmental functions) and that sell a portion or all of the food produced at the site.
- Eight collective gardens characterized by non-commercial purposes on land cultivated by community groups.
- 55 individual gardens that were non-commercial with land divided into plots managed by individual gardeners. These included allotment plots and home gardens.

**Table 1:** Breakdown of case studies by location (city and country) and type of urban agriculture. Growing season duration is measured in the number of days between the last frost in the spring and the first frost in autumn. Temperature refers to the median temperature during the growing season. Weather data came from (NOAA 2019), and demographics data came from (INSEE 2018; Eurostat 2019; U.S. Census Bureau 2019). Values in brackets correspond to the population, density, or years of farm establishment in neighboring, smaller city in the metropolitan area, where some case studies are located.

		Total farms (*n*=72)	Urban farm (*n*=9)	Collective garden (*n*=8)	Individual garden (*n*=55)	Years farms established	Population	Population density (inhabitants/km^2^)	Temperature (°C)	Rainfall (mm)
France	Nantes area	13	2		11	1982 [2016]	309,346 [8,541]	4745 [656]	15.8	787
	Paris area	3	3			2014 [2018]	2,187,526 [86,375]	20,755 [14,996]	16.7	672
Germany	Bochum	2			2	1947	364,920	2510	14.4	841
	Dortmund	2			2	1938	586,600	2087	14.3	698
	Lünen	1			1	1996	86,465	1459	14.3	689
	Münster	6			6	1902 [1922]	313,559	1030	14.3	652
Poland	Gorzów Wielkopolski	35	2		33	1975 [2018]	123,921	1439	14.8	423
Great Britain	London area	5	2	3		2008 [2020]	8,825,000 [29,303]	5575 [4600]	14.4	655
United States	New York	5		5		2013 [2018]	8,622,698	10,636	18.3	1488

Cities had variable populations and demographics (Table 1) but had similar temperate climate characteristics and weather (Beck et al. 2018).

#### France

In France, 16 sites were selected, including 11 individual gardens from an allotment garden association in Nantes and five urban farms (two in the Nantes area and three in Metropolitan Paris). The two urban farms in Nantes are commercial farms with the main goal of producing food (determined through surveys with participants in all case cities). Two other urban farms are school gardens located in Paris, with the main function of education. The last urban farm in Paris focuses on professional integration and training as well as food production. The main goal of the allotment garden site is community cohesion and development. All stakeholders were involved in the project thanks to the network of the French team—no financial incentive compensated their voluntary participation.

#### Germany

In Germany, 11 allotment plots were selected as case studies. They are located in the metropolitan Ruhr area (in the cities of Dortmund, Bochum, and Lünen) and in Münster. Individuals or families use the plots for food production and leisure, and at least one third of the area must be used for the production of food according to German Allotment Law.

The participation of gardeners was organized with the help of the federal allotment garden association (‘*Landesverband der Kleingärtner Westfalen Lippe e.V.*’) through an informative workshop about the research project and the tasks ahead in November 2018. Participants received a small financial incentive afterwards (450€).

#### Poland

The 35 sites examined in Poland are located in Gorzów Wielkopolski, a city in northwestern Poland. Case studies included 2 urban farms and 33 individual gardens. Enrollment for the project was carried out in 2018 with the help of the Polish Allotment Gardeners Association, Gorzów Wielkopolski branch *(‘Polski Związek Działkowców, okręg Gorzów Wielkopolski’)* and the municipality. Gardener/farmer participation was voluntary, without any financial incentives.

All investigated sites are individually managed. The main motivation for gardeners at individual gardens is recreation, but also food production for their own and their families' needs. The two urban farms are run individually, focused on sales at the local market.

#### United Kingdom

Case studies selected in the UK include two urban farms and three collective gardens. All case studies are in the London metropolitan area.

All case studies share social objectives and are connected with local groups and organizing activities to improve wellbeing, or to produce educational activities for local schools. The destination of food harvested varies across all case studies, with the urban farms and collective gardens selling their produce, and the latter also donating food to their volunteers and gardeners.

We selected sites with the assistance of Social Farms & Gardens (SF&G), a UK charitable organization that operates on behalf of community gardens, care farms and urban farms. The team launched a call to all SF&G London-based members, asking for expressions of interest to participate in the project. Researchers visited the 30 interested farms/gardens and partnered with nine sites; five sites collected data of sufficient quality to be included in the study. A small incentive was offered to each participating case study (£100).

#### United States

The US sites consist of six urban farms located within public housing developments in New York City. They are distributed across four of the city’s five boroughs.

These sites are farmed by teams of young adults who are employed and supervised by Green City Force, a non-profit organization that provides workforce training and support to economically vulnerable youth living in public housing. Green City Force staff also provides technical support and labor for the farms. The project’s goals are education and training, food production for free distribution to public housing residents, and ancillary services to the public housing community (e.g., educational tours, community events, cooking and nutrition instruction). Green City Force engaged the urban farmers in collecting operational data for the FEW-meter project as a component of their farm training and to learn about the environmental and social impacts of UA.

### Data collection 

Two main methods were used to collect qualitative and quantitative data: observations by the research team during site visits, and monitoring food production and agronomic inputs by study participants.

#### Site visits and farmer/gardener surveys

Data collection by the gardeners/farmers was accompanied by regular site visits by the researchers. These visits consisted of exchanging information with gardeners/farmers about data collection and reliability, measuring spatial and infrastructure data, and observing the site and reporting significant changes in operation to improve data consistency. The frequency of visits depended on the site and the agreement with the stakeholders.

As part of these exchanges, we surveyed the organizations (e.g., non-profits, government agencies) responsible for supervising our farm and garden sites, as well as individual farmers and gardeners. Four survey items were employed in this analysis: (i) Organizational goals; (ii) Use of time spent in the garden/farm (actively gardening vs other activities); (iii) Importance of access to fresh food; and (iv) Years of experience gardening. Team members developed all survey items from existing literature and pre-tested them with gardeners in Germany. Additional methodological details can be found in Caputo et al. (2021) and Kirby et al. (2021).

#### Monitoring food production and agronomic inputs

To record food production and inputs, gardeners/farmers entered data manually into a printed harvest booklet or used an alternative data recording system if one was in place (e.g., at one site in France, two sites in the UK and all US sites). The gardeners/farmers collected data on: (i) their food production, including harvest date, crop type, quantity harvested in kilograms or other units (which were later converted to kilograms by researchers), and destination of the harvest; (ii) quantity and source of water used for irrigation; (iii) use of electricity and fuels; (iv) fertilizers, soil amendments and pesticides (organic and synthetic) used; and (v) participation in social or educational events.

The layout of the harvest booklet differed slightly among study sites and was adapted to the individual needs of the gardeners/farmers, but the metrics collected were standardized across sites. We provided intensive support for data entry, gave regular feedback, and provided materials such as scales for weighing produce and water meters when necessary.

### Data processing 

Data collected from only March 2019 to October 2019 were included in the analysis. We deemed data from 2020 unrepresentative of a typical growing season due to disruptions from the Covid-19 pandemic. All data collected were gathered in a cloud-based database using Airtable© software. To analyze the primary data, we developed indicators representing food production and resource use.

#### Land allocation

The land allocation per site was composed of three types of land uses:

- Total site area: the administrative boundaries of the project.
- Cultivated area: surface dedicated to cultivation of edible crops and inedible plants or grassy spaces, including pathways within cultivated plots.
- Food production area: area dedicated only to the cultivation of edible crops, as opposed to ornamentals, including pathways within the productive plots.

We then created two relative indicators:

$$\mathrm{Cultivated\; area} \left(\%\right)= \frac{\mathrm{cultivated\; area}\, {(m}^{2})}{\mathrm{total\; site\; area}\, ({m}^{2})}$$

$$\mathrm{Food\; production\; area} \left(\%\right)= \frac{\mathrm{food\; production\; area}\, ({m}^{2})}{\mathrm{cultivated\; area}\, ({m}^{2})}$$

#### Food production

Farmers/gardeners recorded harvests in mass or the number of vegetables (e.g., 3 heads of lettuce). To express food production in a common unit, we standardized all harvest records to mass-based units using conversions supplied by farmers and gardeners (e.g., 1 bunch = 0.1 kg). Based on this measurement and food production area, we calculated the following indicators:

$$\mathrm{Yield} \left(kg/{m}^{2}\right)= \frac{\sum \mathrm{harvest\; weight\; in\; fresh\; biomass}}{\mathrm{food\; production\; area}\, ({m}^{2})}$$

$$\mathrm{Calories\; per\; }{\mathrm{m}}^{2}= \frac{\sum \mathrm{calories\; per\; kilogram\; crop}\, \times\, \sum \mathrm{harvest\; per\; crop}}{\mathrm{food\; production\; area}\, ({m}^{2})}$$

Calories per kilogram of crop were determined using the USDA Food Intakes Converted to Retail Commodities Databases (Bowman et al. 2013) and the USDA Food and Nutrient Database for Dietary Studies (U.S. Department of Agriculture, Agriculture Research Service 2018). When specialized crops were unavailable in one or the other database, we employed proxies (e.g., basil for purple basil).

To assess the cultivated diversity, the number of crops harvested was measured, and we calculated a cropping diversity indicator calculated with the following formula:

$$\mathrm{Crop\; diversity\; per\; }{\mathrm{m}}^{2}= \frac{\mathrm{number\; of\; different\; crops\; harvested}}{\mathrm{food\; production\; area\, }({\mathrm{m}}^{2})}$$

#### Resource use

Fertilizer type and quantity were recorded at each application, and the nitrogen, phosphorus, and potassium (NPK) content for each synthetic fertilizer (percent by mass) was recorded. Using these numbers, masses of each synthetic fertilizer were converted to the NPK values with the following equation:

$$\mathrm{Synthetic\; NPK\; input}=\sum\, \%\, \mathrm{NPK\; by\; mass\; per\; fertilizer\, }\times\, \mathrm{mass\; fertilizer\; applied}$$

Type and quant﻿ity of organic fertilizer and other supplies used (e.g., mulch, pesticide) were also recorded upon application but were not converted to NPK values.

For irrigation water consumption, a measurement system was set up in each study site either through automatic measurement (e.g., using a water-meter) or by designing a measurement system (e.g. tracking the number and volume of buckets or watering cans used). Based on the total measured water use, we calculated irrigation water consumption per square meter of the food cultivated area and per kilogram of fresh biomass.

Farmers and gardeners recorded their electricity and fuel use throughout the growing season. Electricity was tracked using meters, and the volume of fuel consumed by vehicles and equipment was recorded. We converted the volume of fuel to a common unit of kWh to be consistent with electricity using a conversion factor of 9.3 kWh/liter of fuel (U.S. Department of Energy 2021). The energy used from both sources was summed to find the total energy use at the site.

### Data analysis 

We cleaned and clarified the data through direct exchanges between the research team and farmers/gardeners. Eight of the initial 80 sites were excluded from further analysis due to unreconcilable data quality issues, leaving a final sample of 72 farms/gardens. The final dataset is available in supplementary material 1. All analyses were conducted in R (R Core Team 2020).

We prepared descriptive statistics across our sample of farms, describing the crop diversity, resource use, and yield measured in our sample. To explore potential explanations for the variation in results, we tested for correlations among several of our variables and weather characteristics in each city (annual precipitation, duration of the growing season [measured as the number of days between the last and first frost date in 2019], median temperature during the growing season, and average daily hours of sunlight). For all sites, only data from March 2019 through October 2019 were analyzed, regardless of the length of the growing season. Because most of the variables had non-normal distributions, we used Spearman rank correlations.

We also tested the correlations between several measured indicators, such as yield, irrigation water use, and nutrient use. Relationships described in the text as significant had a *p*-value lower than 0.05. ANOVA Tukey’s Honestly Significant Difference tests were used to evaluate the statistically significant differences between countries and types of UA.

Finally, we used a cluster analysis to explore the factors driving yield in our sample. We employed a Euclidean distance clustering algorithm in the base stats package in R, segmenting the dataset based on yield, irrigation water use, compost use, manure use, crop diversity, and individual vs collective management. We analyzed the Within-Cluster Sum of Squared Errors to determine the ideal number of clusters and characterized the resulting three clusters using the segmentation variables as well as several variables from the organizational and farmer/gardener surveys.

## Results and discussion

We found that resource use and yields varied significantly across our sample. Climate and location could not explain most of these differences with any statistical confidence. However, we did find that farm type — collective garden, individual, garden, or urban farm — could explain differences in several dimensions of resource use. For example, we found that UA sites varied dramatically in their allocation of lands to different types of services, as collective gardens prioritized community gathering space, while individual gardens offered more space for private leisure and non-crop biodiversity.

However, this common typology failed to predict production potential on our sites, and the most important differentiator appears to be collective vs individual management. About half of the individual gardens studied are highly productive, a gap we link to farmer/gardener experience and training. Similarly, irrigation water use seems to be routinely lower on individual gardens, and we identify several other avenues of future research, including the role of raised beds in exacerbating irrigation demands and challenges associated with more sophisticated irrigation systems like drip lines. We also find that urban water recycling via UA is disappointingly rare and offer several explanations of this, including material constraints and concerns about urban pests. We clarify these findings in detail below.

### Land use varies across forms of UA

**Fig. 2:**
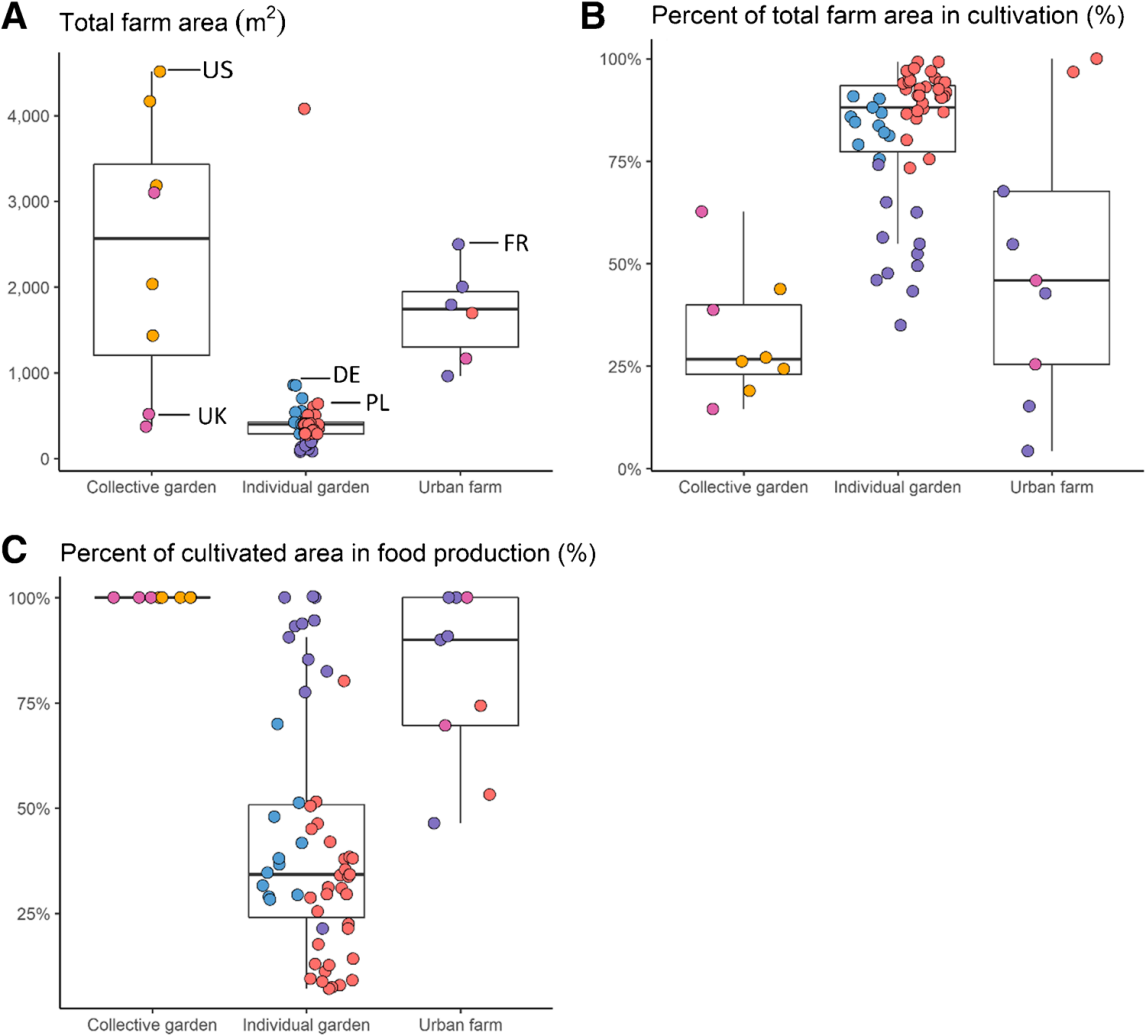
Use of space expressed per (**A**) total farm size, (**B**) percent of the total farm area in cultivation (including inedible plants and other green areas), and (**C**) the percent of area in cultivation that was used for food production. This represents the breakdown of area for non-cultivation, other green spaces, and food production. In part (**A**), three farms with very large areas were removed, and in parts (**B**) and (**C**), two of these three farms were excluded because they also had very large cultivated areas. DE: Germany, FR: France, PL: Poland, UK: United Kingdom, and US: United States.

Across our sample of farms and gardens, the managers cultivated about three-quarters of the area under their control (Fig. 2). This is consistent with other studies, which found average percent area in cultivation in UA ranging from 44 to 76% (Pourias et al. 2015; Gregory et al. 2016; Edmondson et al. 2020). We observed several differences in land use across types of gardens. On average, individual gardens used the highest percentage of their allotted land for cultivation, but they typically used less than half of this cultivated area for food production. Instead, gardeners allocated space to flower beds, hedges, lawn, and biodiversity support. In contrast, collective gardens reserved much more space for non-cultivation purposes like community gatherings and education, but almost all of their cultivated space produced food. This finding has important implications for research and policy evaluating the effects of expanding UA in cities, reminding us that only a fraction of land allocated to gardens may be dedicated to growing food. The differences between community and individual gardens also implies possible tradeoffs in land use between UA’s various services, like community building, food production, and biodiversity provision.

### The quantity of irrigation water used by UA is influenced by growing medium, irrigation system, and cost of water

One of the most popular justifications for expanding UA is the potential for urban symbiosis, or the use of urban wastes to grow food (Goldstein et al. 2016). Despite the potential for non-potable water (e.g., stormwater runoff and wastewater) to irrigate gardens and farms, we found that the most common source of irrigation water in our sample of farms and gardens was municipal drinking water. In fact, municipalities provided almost all the irrigation water used on more than a third of case farms, similar to other studies (Csortan et al. 2020; Whittinghill and Sarr 2021). This is of note, especially for cities with water scarcity or carbon intensive municipal water supplies (Dorr et al. 2021). Groundwater wells were also utilized, particularly at allotment complexes near the smaller cities of Nantes, Münster, and Gorzów Wielkopolski. Rainwater collection was uncommon on our farms and gardens. Past research and personal communication with gardeners suggest that this might be due to material constraints (e.g., space necessary to store water, weight issues on rooftops, the cost of rainwater collection systems) or the perceived health risks of stored water (e.g., mosquito proliferation) (Wilke et al. 2020).

**Fig. 3:**
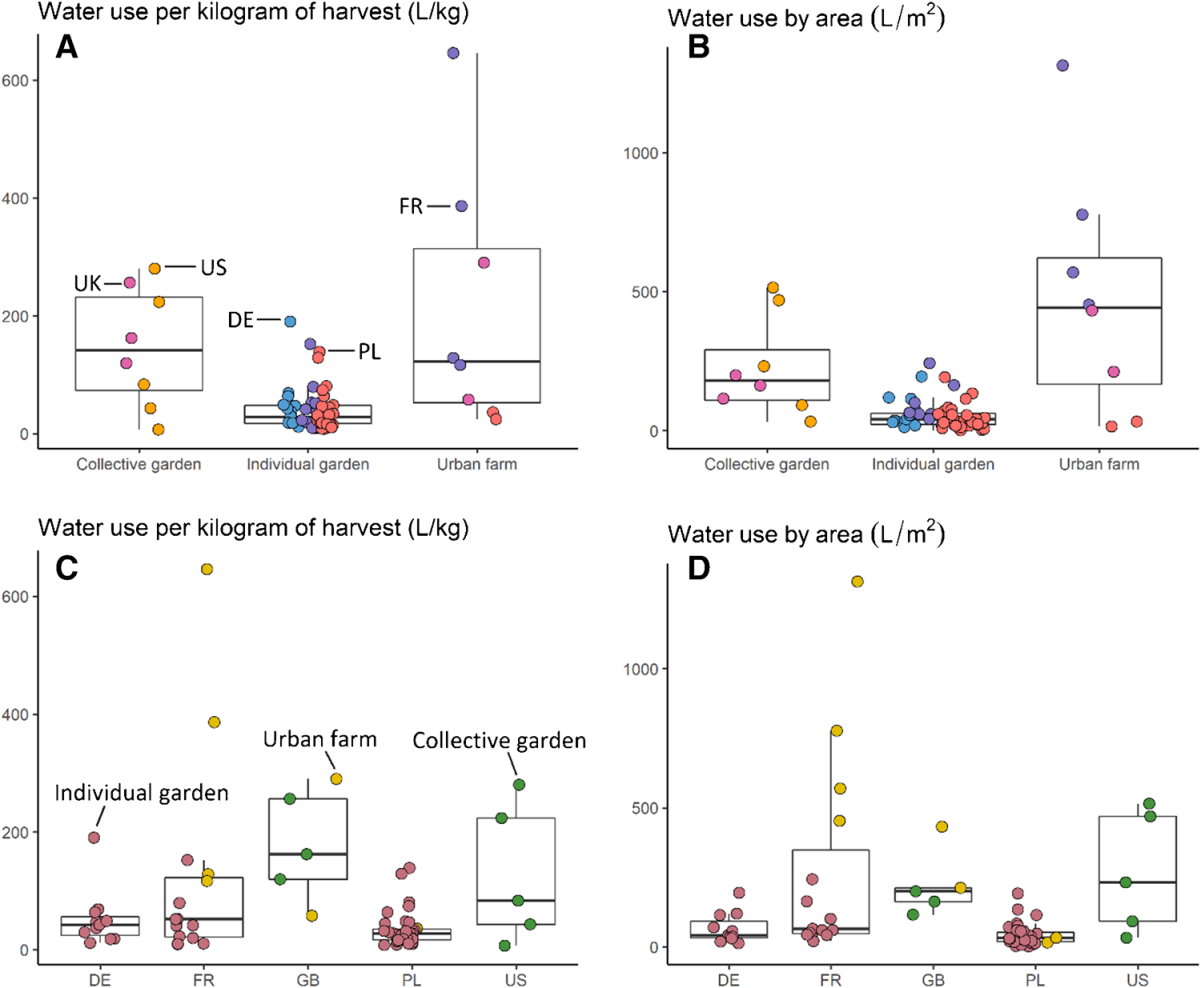
Water use expressed in terms of food produced (**A** and **C**) and of area in food production (**B** and **D**), and compared between types of UA (**A** and **B**) and countries (**C** and **D**). Water use from one French urban farm on an area basis was very large and removed from figures and analyses, and was 2802 L/m^2^. Similarly, on a food production basis, a different French urban farm had very large water use (1942 L/kg) and was removed.

Most farms/gardens studied used between 28.4 and 114 L/m^2^ of area in food production (the first and thirds quartiles), with a mean of 122 L/m^2^ (Fig. 3b, d), although we found several extreme values on either side of this range (from 1.7 to 1313 L/m^2^). Only one statistical outlier was detected and excluded from analysis of irrigation water use — an urban farm in France that used an exceptionally large amount of irrigation water (2802 L/m^2^) due to leaks in the drip irrigation system that was poorly calibrated and excessively used.

There was similarly large variation in irrigation water use based on food production, ranging from 6.9 to 646 L/kg of food, with a mean of 71.6 L/kg (Fig. 3a, c). There were important differences in irrigation water use between types of farms/gardens, where individual gardens from all countries had substantially lower use than the other types. Considering this extreme variation across our sample, and to ensure that our data were valid and reliable, we tested for a relationship between the amount of irrigation water used and the measurement method (e.g., automated water meter vs counting buckets) and we found no relationship.

In terms of frequency, all farms/gardens used irrigation. In contrast, most rural vegetable farms rely only on rainfall. For example, in the USA in 2017, only 21% of vegetable farms used irrigation (USDA 2018, 2019).

There is insufficient data on the quantity of irrigation water typically used by UA to make broad comparisons to our results. Pollard et al. (2018a) pointed out that there are not many articles focusing on the measurement of irrigation water consumption in UA, and Whittinghill and Sarr (2021) found that irrigation was the least-recorded practice among urban farmers and gardeners surveyed. Our results are largely consistent with the largest known survey to-date, a study of 163 allotment plots in the UK (Dobson et al. 2021). While they found a much smaller average than ours overall (16 L/kg vs 71.6 L/kg), their findings were much closer to our average for individual gardens (16 L/kg vs 40 L/kg), which reflects our findings that individual gardens may use water more efficiently than collective gardens or urban farms, or they may simply under irrigate. Dobson et al. also found extreme variation in irrigation; similar to our case studies sites, those that used the most water used substantially more than other sites (Dobson et al. 2021). Another assessment of irrigation based on a large sample of 68 UA systems (Dorr et al. 2021) found that irrigation water use averaged 107 ± 121 L/kg (mean and standard deviation), in agreement with our study. Furthermore, analysis of rural fruit and vegetable crop production in the five countries also reveals substantial variation, with typical irrigation water use between 1.29 L/kg (Poland) and 120.97 L/kg (US) (Hoekstra and Mekonnen 2020). This indicates that while irrigation may be more common in UA, overall irrigation rates are not out of line with those practiced by professional farmers in rural areas.

We identified several avenues of future study based on the irrigation practices observed at our farms and gardens. For example, we found a weak positive correlation between precipitation and irrigation water use per m^2^ (ρ = 0.48, see supplementary materials 4 and 5 for all correlations). While it is counterintuitive that farms/gardens in cities with more rain use more water for irrigation, we hypothesize that in these cities with wetter climates, farmers, and gardeners may cultivate more water-intensive crops or may water excessively, since water scarcity is not a concern. This is consistent with the much better studied field of rural agricultural irrigation, where scholars have long pointed to differences between total rainfall and effective rainfall that plants can use (Hershfield 1964) and many studies describe disconnection between rainfall, weather forecasts, and farmer decision-making about irrigation (e.g., Yoon et al. 1993; Wang and Cai 2009; Muller et al. 2021). Future work should assess urban farmer/gardener crop choices and irrigation scheduling systems across an array of climates and farm/garden types.

Our evaluation of the effects of the methods of irrigation found that more water was used at farms using drip irrigation (mean and standard deviation of 345±289 L/m^2^) than those using hoses (104±201 L/m^2^) or watering cans (74±63 L/m^2^). This was surprising because drip irrigation is promoted as a precise, water-saving irrigation tool. However, drip systems may be more sensitive to lack of professional management and garden inattention. For example, drip systems may use more water because they are prone to leaks, and, since they are often automated with timers, require careful calibration or expensive soil moisture monitors to optimize efficiency for polycultures. Manual watering is more labor intensive and so may be practiced less frequently or for shorter durations than automatic drip irrigation. We also found that farms that did not pay for water irrigated more than those that paid for some or all water (277±364 vs 74±76 L/m^2^). This may have implications for government policies to offer water cost reductions to gardens and farms that conserve or use alternative non-potable sources. Finally, systems growing primarily in soil had lower irrigation water use than those growing in substrate (i.e., built up growing media, often in raised beds but not exclusively) (99±207 L/m^2^ vs 277±188 L/m^2^). Recent work in New York has found that substrate design is important for managing water retention in rooftop settings (Harada et al. 2020), and our results indicate that ground-based raised beds would benefit from similar study and optimization.

### UA relies primarily on organic inputs, though the amount used varies 

**Fig. 4:**
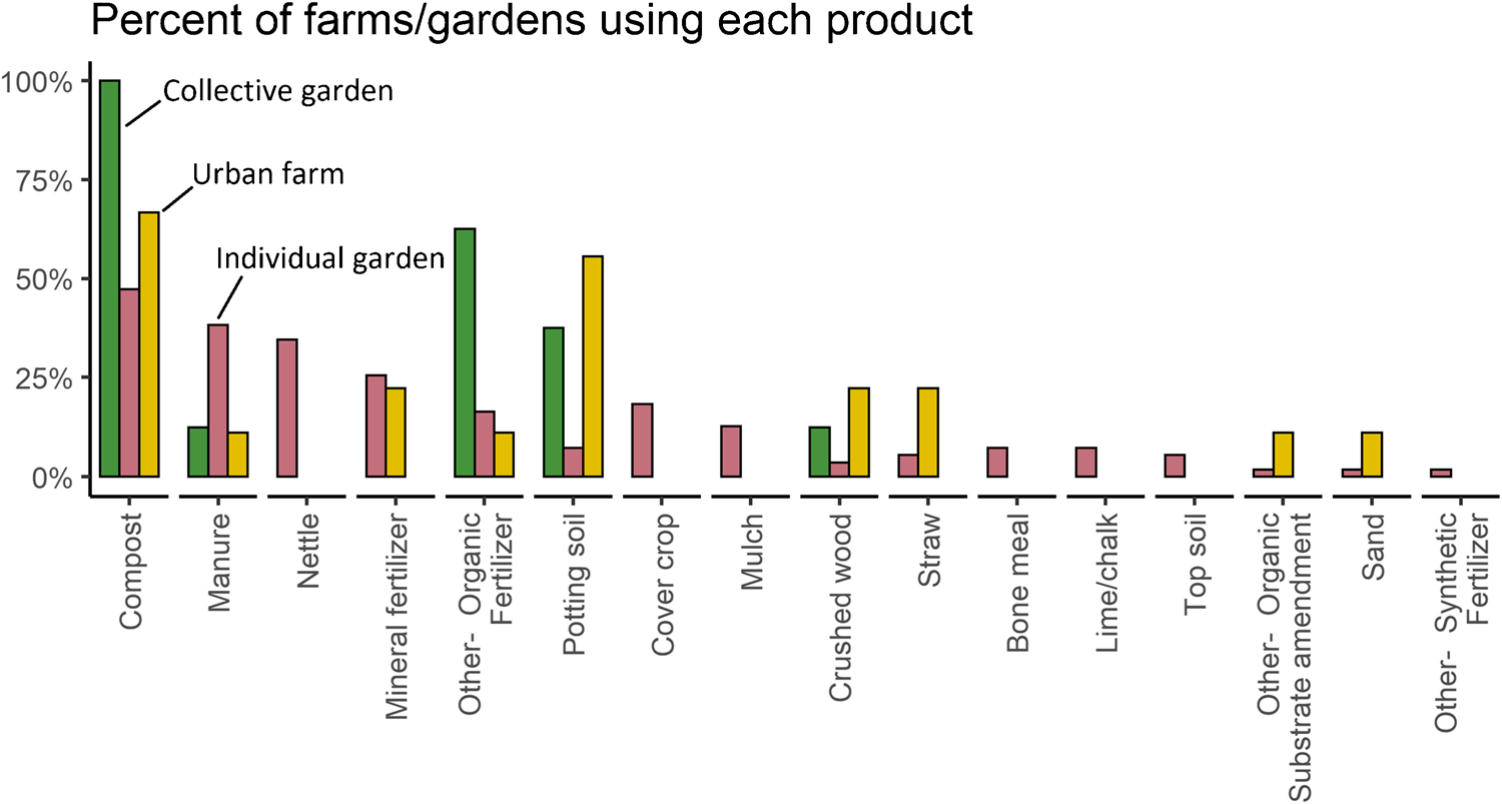
The frequency of use of various inputs is shown for the different types of urban agriculture.

Substrate/soil amendments and fertilizer were used by 94% of the farms/gardens. The most common input was compost, which was used at 52% of farms/gardens (Fig. 4). However, this varied by case study context: while all gardens in the USA and UK used compost, 75% of the individual gardens in Nantes relied on animal manure (only 8% used compost). Sites growing in substrate were more likely than those growing in the soil to use compost (92 vs 44%) and potting soil (50 vs 9%). Overall, manure was the second most common input and was used by 32% of all sites. Manure was much more popular on individual gardens (used on 91% of those sites) and was used more often in smaller cities (i.e., not in London, Paris, NYC), perhaps because of easier access to rural sources or greater risk of complaints over manure odors. In stark contrast to the overwhelming majority of conventional rural farms, mineral fertilizers appeared on only 22% of our sample of urban farms and gardens. Almost half of these mineral fertilizers, including calcium and rock flour application, still qualified as organic cultivation practices. Synthetic nitrogen, phosphorus, and potassium inputs are listed in supplementary material 1.

Pesticides were not used as frequently as soil amendments: only 29% of farms used a pesticide. For 7% of farms this was an organic pesticide, and 24% of farms used a synthetic pesticide (some used both organic and synthetic pesticides). These were mostly synthetic fungicides (used by 19% of farms), insecticides (11%), molluscicides (3%), organic molluscicides (3%), and other organic pesticides (6%). Pesticide use varied significantly by type of farm: 51% of individual gardens, 22% of urban farms, and no collective gardens used them.

Our results are largely consistent with the existing literature on inputs in UA. Numerous surveys of UA have found that compost is the main input and compost production is the main practice, often followed by the use of manure or other organic amendments, with synthetic fertilizers and pesticides used less frequently (Guitart et al. 2012; Dewaelheyns et al. 2013; Gregory et al. 2016; Kirkpatrick and Davison 2018; Pollard et al. 2018b; Wielemaker et al. 2019; Edmondson et al. 2020; Nicholls et al. 2020; Dobson et al. 2021; Whittinghill and Sarr 2021). While it is helpful to understand the types of inputs used by UA, there is a clear gap in the literature when it comes to quantifying the amount of input used on urban farms and gardens. Those that have tracked this have reported that inputs were difficult to measure and have high uncertainty (Wielemaker et al. 2019). It is perhaps not surprising, then, that our results are inconsistent with this limited literature. For example, Dosbon et al. (2021) found an average of 1.9 L compost used/kg food in allotment plots, compared to a mean and standard deviation of 5.5±6.3 L/kg and median of 3.0 L/kg here for the 38 sites that used compost. On an area basis, these sites used an average of 9.7±11.5 L of compost/m^2^, with a median of 4.9 L/m^2^. This important variability of practices and related inputs appear to be linked to several factors that we cannot distinguish within the realm of this study: heterogeneous practices among sites and gardeners, the context (notably the resources accessible) as well as the willingness of farmers to use local inputs. Those factors are similar to those mentioned in others studies on the topic (Dobson et al. 2021; Wielemaker et al. 2019; Whittinghill and Sarr 2021). As reported by Wielemaker et al. (2019), the important use of organic matter for some farms/gardens could lead to an over fertilization of crops. Compost and other substrate amendments can have a significant carbon footprint as well as a direct impact on nutrient management and soil health; this is an important area of future study (Dorr et al. 2021; Wielemaker et al. 2019).

### Energy use on low-input UA is unlikely to impact cities

At 40% of farms/gardens studied, no electricity or fuel was used on-site. The farms that used external energy had a mean and standard deviation of 1.7±3.9 kWh/kg of food, and median of 0.5 kWh/kg of food. Most of the sites had very small energy use, with 90% of values below 2.5 kWh/kg of food. While it is important to measure on-farm energy use, more significant quantities of energy use in UA occur off-farm, such as in the municipal water treatment and distribution process, for transportation of people, produce and material, and for the production of infrastructure and inputs (Mohareb et al. 2017).

Perhaps because of this challenge in scoping, on-farm energy use is not usually measured for the type of urban farms/gardens studied here. This data is generally more available, and arguably more relevant, for controlled environment UA where lighting and temperature control may use substantial amounts of energy (Martin and Molin 2019; Pennisi et al. 2019). Nevertheless, the relatively small energy use documented here is noteworthy because large amounts of energy use are embedded in the global food system, with the food system accounting for an estimated 30% of global energy use (FAO 2011).

### Yield varies in UA, particularly between individual and collective gardens

**Fig. 5:**
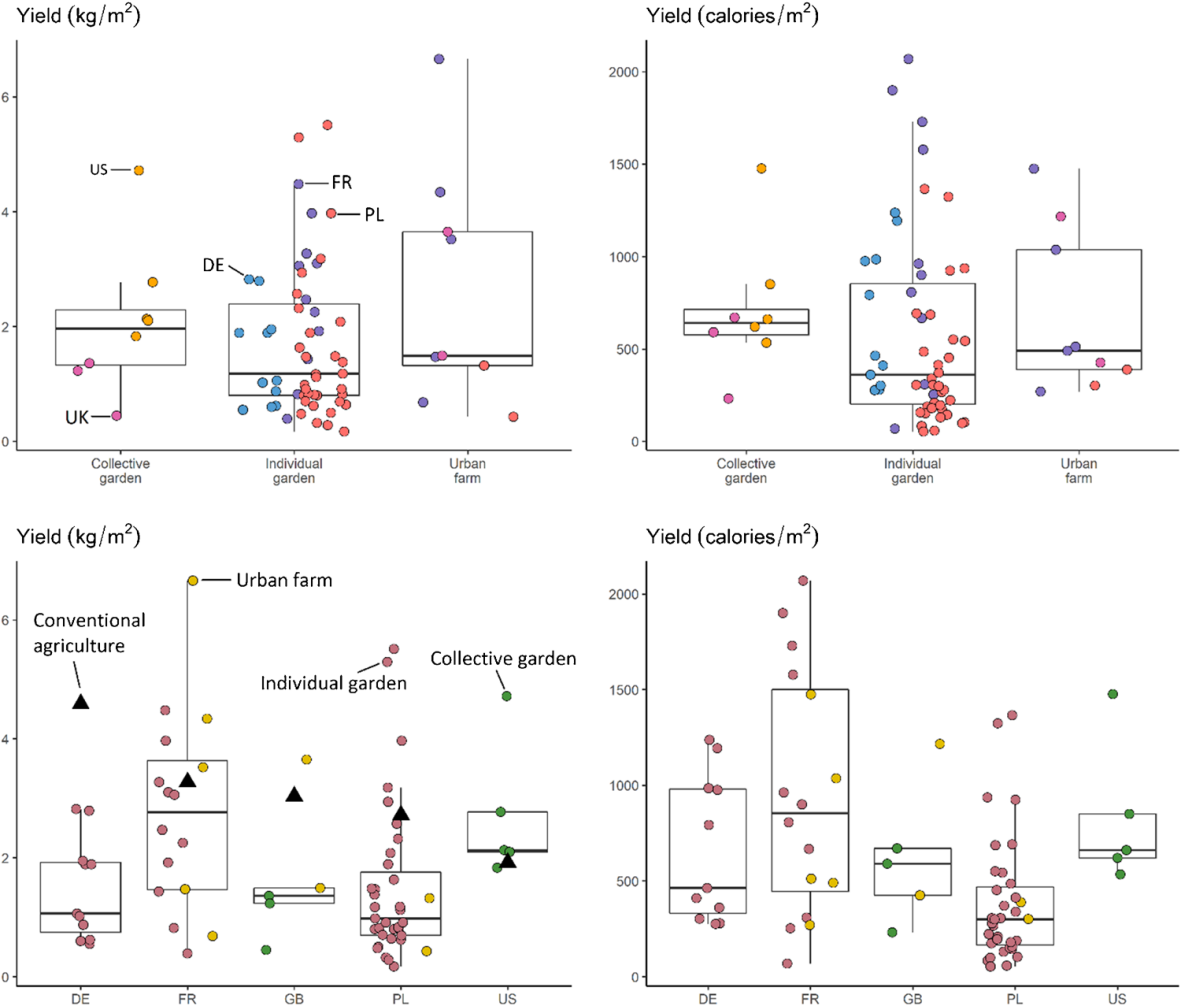
Food production expressed in yield by mass or calories per country or urban agriculture type. Points represent results from individual farms. In the lower left histograms, triangles represent average yields for vegetable production from each country from FAO statistics. For the USA, we used data only from the state of New York, where the case studies were located.

Yield varied from 0.2 to 6.6 kg/m^2^, with a mean and standard deviation of 1.9±1.4, and median of 1.5 kg/m^2^ respectively (Fig. 5a, b). The farm with the largest yield, an urban farm in Nantes, France, was substantially larger than the second largest (6.7 vs 5.5) and was the only case study where all of the production was done in a greenhouse. This was also a commercial farm in which food production was the main objective. The mean and standard deviation for calorie production per m^2^ were 596±477, and the median was 439 (Fig. 5c, d). The maximum value was observed for an individual garden in France with 2069 calories/m^2^, while the minimum value was observed at an individual garden in Poland with 52.8 calories/m^2^.

Our results are consistent with the literature on UA yields. Other studies have reported yields on most urban plots between 0.5 and 2 kg/m^2^ (Gittleman et al. 2012; Smith and Harrington 2014; CoDyre et al. 2015; Sanyé-Mengual et al. 2015; Sovová and Veen 2020; Nicholls et al. 2020; Dobson et al. 2021). Similar to here, other studies report that isolated cases can achieve significantly higher yields, between 3 and 6 kg/m^2^ in community gardens and rooftop farms (Algert et al. 2014; Pourias et al. 2015; McDougall et al. 2019; Appolloni et al. 2021). Exceptional individual gardens and rooftop farms have been shown to produce 10–16 kg/m^2^ (Boneta et al. 2019; Grard et al. 2020; Nicholls et al. 2020). No gardens in our sample achieved this level of productivity.

The UA classification scheme used throughout this paper (individual gardens, collective gardens, and urban farms) is commonly used in the literature, although with no strict definitions or consensus. It is largely based on management structure and crop fate (e.g., personal use, sale). Our results showed that this common classification of farms and gardens was not predictive of yield (Fig. 5). In response, we performed an exploratory cluster analysis on our case studies, segmenting the sample by crop management, harvest, and resource use. Results revealed that collective farms and gardens (both collective gardens and urban farms in the original typology) appear quantitatively similar (Table 2). Individual gardens, on the other hand, cluster into two groups roughly correlating with intensity of food production.

**Table 2:** Cluster analysis based on food production and resource use revealed farm/garden groupings that differed from the common urban agriculture typology we used for much of the analysis in this paper. Urban farms and collective gardens were grouped together in cluster 1, and individual gardens were split into two clusters: those oriented towards food production (cluster 2) or towards leisure (cluster 3). *Three farms were excluded from the cluster analysis because we lacked proper survey data on motivations, experience, and time allocation in garden.

*Farm/garden characteristic*	Cluster 1: collective farms and gardens	Cluster 2: production-oriented individual gardens	Cluster 3: leisure individual gardens
Number of farms/gardens*	17	19	36
*% individual gardens in the cluster*	0%	100%	100%
*Harvest (kg) per m*^*2*^	2.36	2.57	1.24
*Irrigation water use (L) per m*^*2*^	495.2	85.8	39.8
*Compost use (L) per m*^*2*^	13.5	3.7	1.8
*Manure use (L) per m*^*2*^	0.13	3.28	0.61
*Crop diversity per m*^*2*^	0.07	0.29	0.10
*Years of experience farming/gardening*	9.26	30.71	23.12
*% of site in food production*	35%	30%	32%
*% of organizations who list food production as primary goal*	24%	26%	69%
*% of hours in garden spent gardening*	75%	60%	64%
*Average rating of importance of access to fresh food*	4.4/5	4.8/5	4.6/5

While there is a clear tendency for gardens in our second cluster (production-intensive individual gardens) to invest more resources into food production, our attempts to measure gardeners’ focus on food production failed to capture this variation. Of the motivation and management variables assessed, only ‘Years of experience farming/gardening’ clearly reflected the distinction between clusters of individual gardens, and we cannot determine causality (i.e., whether more experienced gardeners are more productive or whether more productive gardeners tend to stick with gardening longer). Still, the emergence of ‘Years of experience farming/gardening’ as a meaningful differentiator between clusters indicates that horticultural skill may be an underlying variable driving the difference across individual gardens.

In fact, this explanation is consistent with previous research on UA. UA with professional and highly trained gardeners or farmers produces 5.4–7.1 kg/m^2^, according to a review by Weidner and Yang (2020). We also found the influence of professional management when comparing UA to rural vegetable production. In three of five case study regions, we found that UA produced significantly less food per unit area than a typical rural vegetable farm (USDA National Agricultural Statistics Service 2017; Food and Agriculture Organization of the United Nations 2022). Only NYC gardens outperformed typical rural farms in their area, while French UA was indistinguishable from rural agriculture. This may be a reflection of the higher proportion of experienced or carefully trained managers in our French and American case studies. In France, all individual gardens fell into cluster 2, with an average of 36 years of experience per gardener. In the USA, all gardeners are members of an AmeriCorps professional development program, where they are carefully trained and supervised by professional farmers.

Our work lays the groundwork for more advanced study of urban garden resource use outside of the laboratory. Future work should consider individual and collective gardens separately and develop larger samples of each type of garden, allowing for more robust analysis of the factors driving yield in urban gardens as there might be other motivations. Furthermore, future studies should focus on farmer/gardener experience and training while also developing new measures of garden/farm focus on food production.

### UA is an important source of crop and flora diversity

The number of different crops grown per farm/garden varied from 1 to 83, with 128 different crops recorded in the dataset. On average, 20±16 crops were grown per farm/garden, with a median of 16. Other studies suggest similarly high, but variable, levels of crop diversity in UA, ranging from 5 to 43 (Kirkpatrick and Davison 2018), 6-36 (Pourias et al. 2015), 28-54 (Grard et al. 2022), and 18–70 (Gregory et al. 2016).

**Fig. 6:**
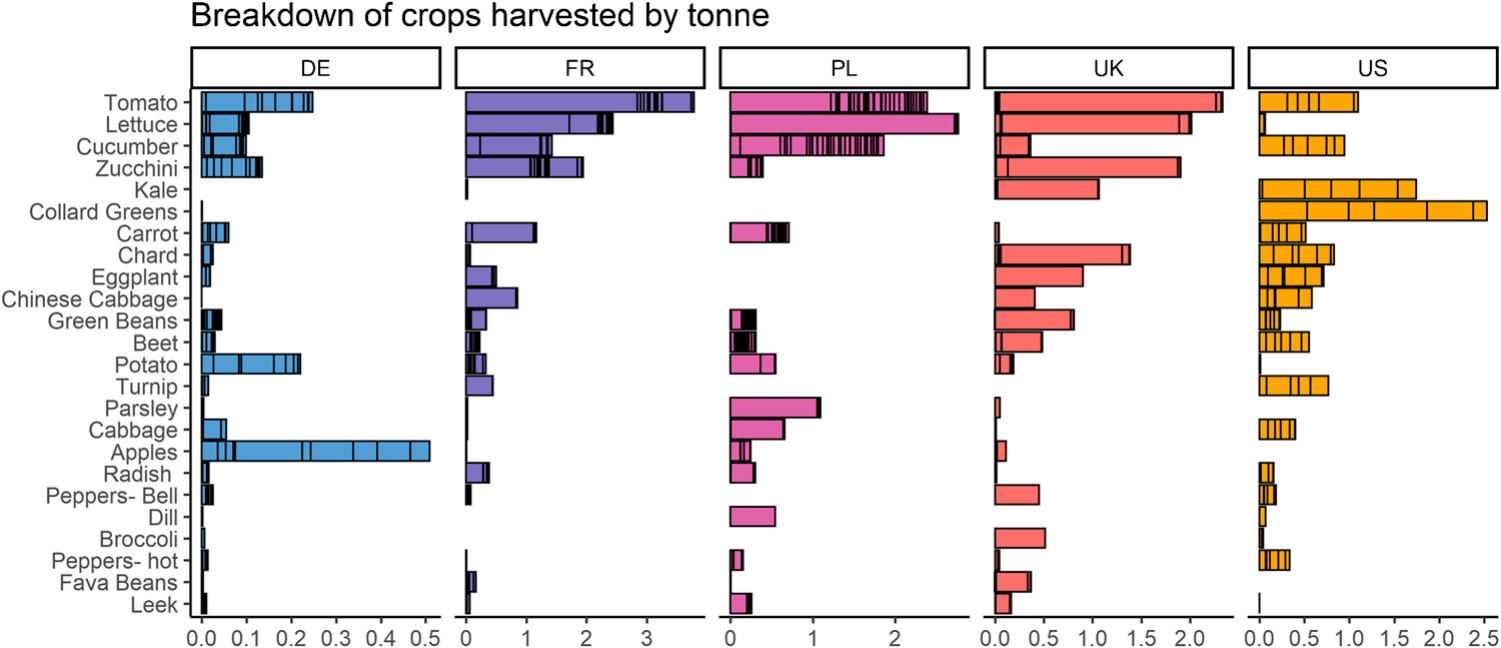
The breakdown of mass of crop harvested per country shown for the top 25 crops. This represents 90% of all harvest by mass. The y-axis shows crops in order of their mass harvested among all farms in the dataset: tomato had the largest harvest, followed by lettuce and cucumber. The black lines within bars represent harvest per farm, where large segments show farms with large harvests. Note in France, Poland, and the UK, one large farm dominated production for crops such as tomato and lettuce.

Twenty-three crops (18% of all crops grown) were only grown at one site. Ten crops appeared at more than half of the farms/gardens. The most common crops in our sample were also the most common crops reported in the UA literature, including tomatoes, cucumbers, beets, carrots, onions, and lettuce, among others (Algert et al. 2014; Gregory et al. 2016; Nicholls et al. 2020; Grard et al. 2022). As has been documented extensively, culturally significant crops were far more popular in particular cities (Taylor and Lovell 2015; Taylor and Ard 2015), such as collard greens observed in our New York gardens (c.f., Fig. 6 below, Gregory et al. 2016). Supplementary material 2 lists the frequency of all crops, while Supplementary material 3 lists the crop names considered synonymous for this analysis and their Latin, scientific names. In addition to crops, about half of the sites grew flowering plants, shrubs, or had native biodiversity areas. Overall, our findings confirm that urban farms and gardens can be important sources of cultivated and non-cultivated flora, and as such support increased urban biodiversity.

### Study challenges

Most limits to this study were tied to its participatory research approach. Because we could only include the sites that were willing to engage with us and collect the data, the case studies are not necessarily representative of UA in each city/country, and categories split unevenly across countries. This is the reality of much case-based UA research (CoDyre et al. 2015). Also, several measurements had large uncertainties because they were self-reported. There were slight differences in our attempted systematic methodology of measurement, especially for input use such as amount of compost and fertilizer, which farmers said was difficult and time-consuming to measure. Farmers/gardeners reported difficulties with consistently reading and resetting water meters, which also led to lost data and early uncertainty. Other studies highlight the difficulty in collecting accurate measures of water use in UA (Pollard et al. 2018a; McDougall et al. 2019).

Another limitation was that we only considered one growing season due to the COVID-19 pandemic and therefore did not assess intra-site temporal variability. Indeed, UA sites regularly experience large changes between years due to weather, changes in farm managers or gardeners, and changes in operation due to shifting objectives or other factors.

The citizen science approach was time-consuming both for the gardeners/farmers who were asked to record their practices, and for researchers who dedicated large amounts of time to fill in missing data, convert between units or data types, and follow up with questions for farmers/gardeners. Indeed, urban farmers and gardeners do not typically collect data about their harvests and input use (Whittinghill and Sarr 2021). We adapted data collection methods, allowing farmers/gardeners to submit data in several different units and types of measures, and obliging researchers to make sometimes complex conversions. For example, compost was measured continuously or annually using units of m^3^, L, and kilograms. Data were collected through tracking regular applications, evaluating purchase records, and measuring the amount of compost made. The complexity of measuring just one input poses an issue for simplified tools aiming to help farmers/gardeners collect data, such as Farming concrete or the Harvest-ometer (Caputo et al. 2021), because they must be adapted to different preferences and data input types. Our hands-on, flexible approach that mobilized many researchers allowed us to collect a unique and large dataset through active partnership with gardeners and farmers.

### Future research

Our results provide a comprehensive accounting of material inputs and outputs from UA sites in five countries. Our analysis raised a number of pressing research questions that future work must address in order to better understand the practice of UA and to support its sustainable growth:

1. What is the resource intensity of individual crops grown using UA? Material accounting exercises of UA, including ours, often look at the farm level. More granular analysis to identify the inputs for a given crop will help identify which crops can be grown using the least resources and show when UA has material advantages over conventional agriculture. This will require either more detailed (and laborious) data collection, studies of monoculture urban farms, or both.
2. What are the environmental impacts of crops grown using UA? Resource intensity (e.g., kWh/kg food) is not equivalent to environmental intensity (e.g., kg carbon dioxide/kg food). Extending material accounts using environmental footprinting methods, such as life cycle assessment, can account for the embodied environmental impacts in UA production. This has been performed for limited case studies using small numbers of farms. Broader studies covering large sample sizes, types of farms, and location are needed to more fully assess the sustainability of UA.
3. What are the resource profiles of different forms of UA or UA in different climates? This study covered a limited cross-section of types and locations of UA. Future large studies should include more intensive forms of UA, such as plant factories, hydroponic greenhouses, and vertical farms. UA is also a global phenomenon. Studies should include farms beyond the Global North and also include a wider cross-section of climates to better clarify its influence on resource intensity (e.g., irrigation use in drier climes).
4. How can UA better leverage residual resources in the city? We found limited application of urban symbiosis — harvesting proximate water and energy — in our case farms apart from reuse of local food waste in the form of compost. Future work should identify barriers — technical, policy, and behavioral — to material recycling in UA.
5. What other factors (social, technological, ecological) have a strong influence on yield and resource use in urban agriculture? For example, future work should explore the effects of expertise among farmers and gardeners, the effects of emotional, time and monetary investment in food production, and the effects of the ecosystem in which a farm or garden is embedded, such as pollinator availability.
6. What drives counterintuitive results in our study? We found that irrigation and rainfall rise together. We hypothesize that this is due to the choice of crops, but other factors, including farmer experience and equipment could be important. Interviews, direct observation, and water meters are needed to explore this further. Multi-year studies would also confirm if this was an anomalous result.
7. How does UA perform in other geographies and climates? We focused on cities in Europe and the USA, with relatively similar temperate climates and socio-economic conditions. It is well known that UA in the Global South has different goals than in our case study locations: How does that affect resource consumption and food production? What about different climates, such as arid or tropical regions?

These are only seven initial questions spurred by our findings. Given the continuing growth of UA, continued research on the potential material and energy implications of this growth is necessary. The short supply chains of UA are often equated with environmental sustainability. Comparing resource efficiency in our sample to other contexts shows that this may not always be the case. The more we understand the conditions for sustainable urban food production, the better academics can support policy makers and farmers in realizing sustainable urban food systems.

## Conclusion

UA is a type of land use that is rapidly growing in cities across the global north. It is, therefore, imperative that researchers and decision-makers develop holistic perspectives on the impacts of these spaces on cities. UA offers a number of social goods and ecosystem services including the provision of fresh, local food. However, these services vary significantly across different types of UA, and this variation and what drives it are poorly understood. We address this gap by studying a large, diverse sample of urban farms and gardens to generate a uniquely powerful dataset for understanding the relationships between farm/garden yields, resource use, and farmers/gardeners.

Analysis of this dataset uncovered a number of trends that should inspire further inquiry. As UA continues to be an important part of cities’ sustainability and resilience planning, a clear understanding of the varying forms of UA will be essential, especially given competing demands for urban land and increasing evidence that not all UA is environmentally beneficial. We confirm that UA’s food production potential and resource requirements are likely to vary significantly across sites and across types of farms and gardens, and while we offer several preliminary explanations for this variation, substantial research remains to be done. In addition to sharing descriptive findings and a unique dataset, this work also demonstrates the utility of a citizen science approach for studying the material inputs and outputs of UA. By working closely with the individuals implementing these practices in cities, it is possible for researchers to better understand what drives the environmental performance of UA. Ultimately, such research is vital for supporting policymaking that enables UA that is good for cities, citizens, and sustainability.

## Supplementary Information

Below is the link to the electronic supplementary material.

Supplementary file1 (XLSX 61 KB)

Supplementary file2 (XLSX 12 KB)

Supplementary file3 (XLSX 19 KB)

Supplementary file4 (XLSX 11 KB)

Supplementary file5 (PNG 44 KB)

## Data Availability

If this paper is accepted, the datasets generated during and/or analyzed during the current study will be available in a public repository.

## Code availability

Not accurate for this study.
