# Supplementary figures and images for: Food production and resource use of urban farms and gardens: a five-country study
Source: Agron Sustain Dev. 2023 Feb 1;43(1):18. doi: 10.1007/s13593-022-00859-4 (PMC9891751; doi:10.1007/s13593-022-00859-4)

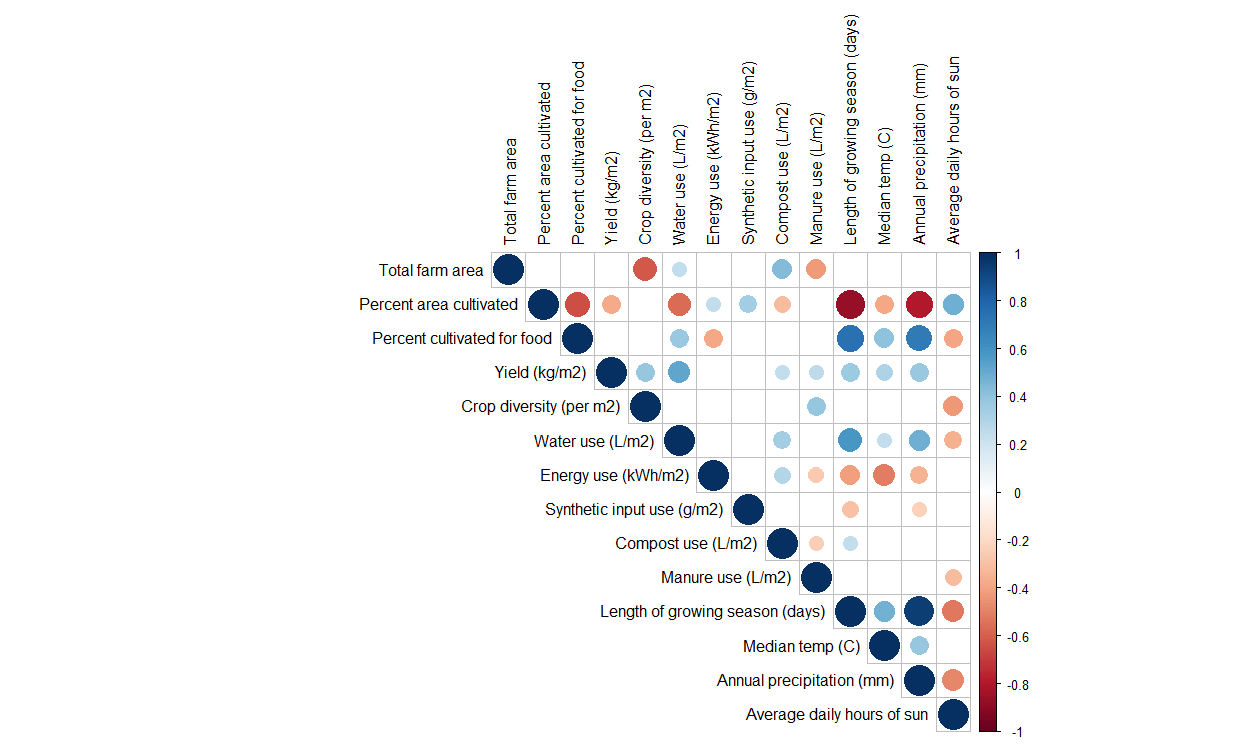

Supplement: Supplementary file 5 — Supplementary file5 (PNG 44 KB) [file 13593_2022_859_MOESM5_ESM.png]
